# Supplementary material for: Chromothripsis during telomere crisis is independent of NHEJ, and consistent with a replicative origin
Source: Genome Res. 2019 May;29(5):737–49. doi: 10.1101/gr.240705.118 (PMC6499312; doi:10.1101/gr.240705.118)
Supplement: Supplemental Material [file supp_gr.240705.118_Supplemental_file_1.zip › contigs/annotated_contigs/DB57/contig.2.DB57_length_405_mean_cov_7.14074074074.docx]

**DB57_length_405_mean_cov_7.14074074074**

```
TCCCACTGCCTTGGGAACGAGATAATCAAACCAATTAGAGCTATATGTAAAATGAAAGTAAAATAGGCCTGAAGGGCTGGCCCACAAGG
 >chr9:71689769-71690066 - E=2e-164 p=3e-03
ACCCCATAAGAGCCCAGATTCCTTAACTTCCACTTTCATGTCAGAGCAGCCCCACTGCATACCTCTTTTGTGCTGTGTCCCAGATAAAG

GAGTACATTATGTCACCGAGTTCTAAGTAAGTGGGGTGCACTGAAACTTTCAGTAGAAGCAAGATGCAAACACATACAAACATGTATGA

TGTTATTTATTTTCTCACTGGCACTG|GCCT|CCCAAACTGGGATTACAAACATGTGCCACCACGCCCAGCTAATTTTTCTGGGGTTTT
                           >chr9:71781945-71782053 - E=2e-53
TTTTTTCGTAGAGACGGGGTTTCACCATGTTGGCCAGGCTGGTTTTG|TACT
```
